# Defining stopping criteria for ending randomized clinical trials that investigate the interruption of transmission of soil-transmitted helminths employing mass drug administration

**DOI:** 10.1371/journal.pntd.0006864

**Published:** 2018-10-01

**Authors:** Marleen Werkman, Jaspreet Toor, Carolin Vegvari, James E. Wright, James E. Truscott, Kristjana H. Ásbjörnsdóttir, Arianna Rubin Means, Judd L. Walson, Roy M. Anderson

**Affiliations:** 1 The DeWorm3 Project, The Natural History Museum of London, London, United Kingdom; 2 London Centre for Neglected Tropical Disease Research (LCNTDR), Department of Infectious Disease Epidemiology, St. Mary’s Campus, Imperial College London, London, United Kingdom; 3 Departments of Global Health, Medicine (Infectious Disease), Pediatrics and Epidemiology, University of Washington, Seattle, Washington, United States of America; Swiss Tropical and Public Health Institute, SWITZERLAND

## Abstract

The current World Health Organization strategy to address soil-transmitted helminth (STH) infections in children is based on morbidity control through routine deworming of school and pre-school aged children. However, given that transmission continues to occur as a result of persistent reservoirs of infection in untreated individuals (including adults) and in the environment, in many settings such a strategy will need to be continued for very extended periods of time, or until social, economic and environmental conditions result in interruption of transmission. As a result, there is currently much discussion surrounding the possibility of accelerating the interruption of transmission using alternative strategies of mass drug administration (MDA). However, the feasibility of achieving transmission interruption using MDA remains uncertain due to challenges in sustaining high MDA coverage levels across entire communities. The DeWorm3 trial, designed to test the feasibility of interrupting STH transmission, is currently ongoing. In DeWorm3, three years of high treatment coverage—indicated by mathematical models as necessary for breaking transmission—will be followed by two years of surveillance. Given the fast reinfection (bounce-back) rates of STH, a two year no treatment period is regarded as adequate to assess whether bounce-back or transmission interruption have occurred in a given location. In this study, we investigate if criteria to determine whether transmission interruption is unlikely can be defined at earlier timepoints. A stochastic, individual-based simulation model is employed to simulate core aspects of the DeWorm3 community-based cluster-randomized trial. This trial compares a control arm (annual treatment of children alone with MDA) with an intervention arm (community-wide biannual treatment with MDA). Simulations were run for each scenario for both *Ascaris lumbricoides* and hookworm (*Necator americanus*). A range of threshold prevalences measured at six months after the last round of MDA and the impact of MDA coverage levels were evaluated to see if the likelihood of bounce-back or elimination could reliably be assessed at that point, rather than after two years of subsequent surveillance. The analyses suggest that all clusters should be assessed for transmission interruption after two years of surveillance, unless transmission interruption can be effectively ruled out through evidence of low treatment coverage. Models suggest a tight range of homogenous prevalence estimates following high coverage MDA across clusters which do not allow for discrimination between bounce back or transmission interruption within 24 months following cessation of MDA.

## Author summary

Soil-transmitted helminths (STH) remain prevalent in many African, South American and Asian countries. The WHO recommends treating pre-school aged children (pre-SAC) and school-aged children (SAC) in areas with endemic STH infections. However, treating pre-SAC and SAC only is unlikely to achieve STH transmission interruption, as adults remain infected and contribute to re-infection in the community, particularly with hookworm. Treating the entire community with high-coverage and high-frequency mass drug administration (MDA) may achieve transmission interruption and ongoing community-based clinical trials are investigating the feasibility and impact of such an approach. Since large-scale trials are costly and STH prevalence may bounce back quickly to pre-treatment levels when MDA ceases, it may be more efficient to stop the trial when interruption of transmission can be ruled out. In these circumstances it may be necessary to investigate why transmission interruption was not achieved to inform necessary improvements in future community-wide treatment programmes. Factors that may affect the likelihood of transmission interruption include water, sanitation and hygiene (WASH), sociodemographic, migration and environmental factors. These factors should be investigated in cases where interruption of transmission is not achieved. In this study, we investigate the stopping criteria required to determine whether transmission interruption has occurred following multiple rounds of MDA.

## Introduction

Soil-transmitted helminths (STH) remain prevalent in sub-Saharan Africa, Asia and South America. STH comprise four main species; whipworm (*Trichuris trichiura*), roundworm (*Ascaris lumbricoides*) and two hookworm species (*Necator americanus* and *Ancylostoma duodenale*) [1]. Throughout the last decade, the World Health Organization (WHO) has focused on the control of STH-associated morbidity in children. One of the WHO 2020 goals is to treat 75% of pre-school and school-aged children (pre-SAC and SAC, respectively) between 2 and 15 years old using mass drug administration (MDA) [2]. However, treating only children is unlikely to result in elimination of STH in many transmission settings, especially if hookworm is the dominant infection, since most of these parasites are harboured by adults. As such, only targeting a small fraction of the infected community results in the requirement for ongoing MDA for morbidity control [3–6]. If interruption of transmission can be achieved, cost savings will be observed in countries that can phase out STH MDA programs and drug donations by pharmaceutical companies could cease [7,8]. At present, the possibility of broadening treatment coverage to include adults for STH (in line with the lymphatic filariasis MDA programme), with the longer-term DeWorm3 goal of interrupting STH transmission beyond 2020 using MDA alone is being investigated.

STH species are dioecious, reproduce sexually within the human host and are thought to be polygamous [9,10]. Thus, to sustain the transmission cycle there must be at least one worm of each sex within a human host to produce fertile eggs. When low prevalences are reached, the likelihood of having both sexes within the same individual decreases [4,6]. If prevalence is very low (the precise level depends on worm aggregation in the host population [9]), a breakpoint will be reached at which reproduction becomes unsustainable and transmission ceases. Consequently, a transmission breakpoint (i.e. interruption of transmission) can be achieved where no further MDA is required. However, both predisposition to heavy worm burdens and non-adherence to treatment may hinder the interruption of transmission [11].

From the 1970’s, several countries (including South Korea) scaled up their MDA programmes (SAC) and prevalence was reduced to less than 0.1% for all STH species by 2004, though this coincided with economic development and hygiene improvement [8,12]. Such improvements are slow to materialize in many countries that currently have endemic STH infections [13,14]. Mathematical models have illustrated that the interruption of transmission is possible with MDA alone, provided that both high coverage and compliance with treatment over multiple rounds are achieved across all age groups in a community (i.e. “intensified treatment”) [3,4,6,10]. However, whether interruption can be achieved with intensified treatment alone and if the high coverage levels needed to achieve interruption of transmission can be achieved in the field is unconfirmed at present. Therefore, community-based trials are ongoing to investigate the feasibility of transmission interruption through MDA-only regimens. These trials include the DeWorm3 project funded by the Bill and Melinda Gates Foundation [15].

With intensified treatment the measured prevalence of infection in a population decreases. However, the likelihood of transmission interruption varies between settings due to stochastic processes, including the intrinsic transmission potential (i.e. the R_0_ value prior to treatment), contact with contaminated soil, worm survival or the egg reduction rate induced by antihelminth drugs [6,16]. In addition, migration patterns could also reduce the likelihood of transmission interruption as it could result in new infections [6]. As such, infections may still bounce back after stopping treatment even when high MDA coverage is achieved.

Clinical trials often use defined stopping criteria when ending a trial early. The progress of the trial is usually monitored by a Data Safety Monitoring Committee (DSMC), which consists of external experts that can recommend termination of the clinical trial based on pre-defined stopping criteria. A clinical trial can be ended before the planned end date as a result of the observed efficacy of the treatment (benefit), evidence of adverse effects (harm), or likelihood of failing to reject the null hypothesis (futility). For the DeWorm3 studies, futility may be the most relevant factor (due to failing to achieve high MDA coverage), but drug safety and efficacy are also closely monitored.

Truscott et al., (2017) illustrated the importance of timing with regards to measuring the prevalence of infection after the cessation of MDA [4]. Statistical confidence in predicting whether interruption of transmission has been successful, was greatest when sampling was done 18–24 months post-treatment cessation. If interruption is not achieved, infections can quickly bounce back to pre-treatment levels. Therefore, determining whether a transmission breakpoint has not been reached at early stages of the trial will be beneficial to reduce efforts of continuous monitoring in settings where transmission interruption is unlikely and to avoid bounce-back to pre-treatment prevalences and worm-burdens during the no-treatment phase [4]. In this study, we investigate whether trial stopping criteria can be defined with confidence in situations where the interruption of transmission is unlikely, based on the prevalence measured six months after the last round of MDA, using an individual-based stochastic model of transmission and treatment.

## Methods

We employed a stochastic, individual-based model of transmission and drug treatment (for both hookworm (*N*. *americanus*) and *A*. *lumbricoides*), to simulate events in the DeWorm3 cluster-randomised trials [17]. The DeWorm3 trials take place in countries with historic lymphatic filariasis (LF) programmes and build upon the MDA treatment provided for LF. One of the drugs used for LF treatment is albendazole, which is also one of the most effective drugs to treat STH [18]. Therefore, countries with a historic LF programme provide good options to investigate the possibility of STH transmission interruption, as past treatment will already have reduced infection levels if high coverage levels have been sustained [19,20].

The model simulates the number of worms present in each individual person in a village over time. Clusters are then constructed randomly from the simulated villages [21]. Individuals contribute to and can acquire infections from the environmental reservoir of infective stages (eggs or larvae). The transmission parameters are age- and species-dependent. For hookworm, adults often harbour the highest worm burdens, whilst children typically have the heaviest worm burden for *A*. *lumbricoides* [22]. The number of worms in an individual follows a negative binomial distribution, i.e. a large proportion of the population have a few worms and a small proportion of the population have many worms. The relationship between the prevalence and mean worm burden for the negative binomial is described by the following equation:

$$P=1-{\left(1+\frac{M}{k}\right)}^{-k}$$

where *P* represents the proportion of individuals infected within a cluster, *M* reflects the mean worm burden and *k* is the negative binomial parameter whose inverse defines the degree of aggregation of the worms. Further details of the model can be found in Truscott et al., (2016) [16]. In this study, we set *k* to 0.285 for *A*. *lumbricoides* [23] and to 0.35 for hookworm [24], these parameter estimates were derived from field studies in Kenya and Zimbabwe. The other parameters are listed in Table 1.

**Table 1:** Epidemiological parameters for *A*. *lumbricoides* and hookworm.

Model parameter description	*A*. *lumbricoides*	Hookworm
Transmission rate (R_0_)	1.6–3.0	1.6–3.0
Aggregation of parasites in hosts (*k*)	0.285 [23]	0.35 [24]
Relative exposure and contribution to the reservoir by age group (assuming no difference between males and females)	- 0–5 years old: 1.61 - 5–10 years old: 1.54 - 10–20 years old: 1 - 20–30 years old: 0.9 - 30–45 years old: 0.59 - 45+ years old: 0.5 [27]	- 0–2 years old: 0.03 - 3–5 years old: 0.09 - 5–15 years old: 1 - 15+ years: 2.5 [26]
Average worm lifespan	1 year (assuming an exponential distribution) [28]	2 years (assuming an exponential distribution) [28]
Female worm fecundity	0.07 [16]	0.02 (assuming exponential saturation) [27]
Reservoir decay rate	mean = 2 months [27]	mean = 12 days [28]
Drug efficacy (egg reduction rate)	0.99 [29]	0.948 [29]
Sensitivity diagnostics (PCR)	0.94 (fertilized and unfertilized eggs) [25]	0.94 (fertilized eggs only) [25]

The DeWorm3 trial compares two arms, including a control arm receiving standard-of-care deworming (in the model, this is reflected as annual treatment of pre-SAC and SAC with MDA) and an intervention arm (in the model reflected as community-wide biannual treatment with MDA). Prior to the start of the trial, at least four years of annual community-based LF treatment has taken place. We assume the following LF coverage levels: pre-SAC = 65%; SAC = 65%; Adults = 40% in both arms over four years. For the follow-up STH treatment, we assume that in the control arm annual coverage levels are 80% in pre-SAC and SAC and that no treatment is provided to adults. For the intervention arm, we assume that the desired treatment coverage of 90% in pre-SAC and SAC and 80% in adults is achieved. Mathematical models, fitted to cross-sectional data of infection prevalence and intensity, predict that these coverages are necessary to achieve transmission interruption over three years of MDA. However, achieving these high coverage levels is difficult, especially in adults. Therefore, we also randomly vary the coverage levels between 50–90% for pre-SAC and SAC, and 40–80% in adults, such that adults have a 10% lower coverage than pre-SAC & SAC. Both arms undergo their DeWorm3 MDA STH treatment for three consecutive years. In the latest scenario, the coverage levels are varied for each simulated village but are then fixed for the programme duration.

In this study we define coverage as the proportion of targeted individuals participating in the MDA treatment. Modelled adherence to treatment can take two extreme forms: random adherence, where at each round the likelihood of taking part is assigned at random, or systematic non-adherence, where individuals either take part in all the rounds or none of the rounds. For example, if the coverage is 75%, then 75% of the individuals participate in each round of MDA, whilst 25% never take part. In this study, we assume a third pattern where individuals comply in a semi-systematic manner which lies between random and systematic adherence. The likelihood of participating in any given round of MDA can be described as *a*_*i*_^*(1-C)/C*^, where *C* represent the coverage in a round of MDA and *a*_*i*_ is a vector of random numbers drawn from a uniform distribution [0,1] assigned to each individual in a cluster. The value of the parameter *a*_*i*_ can be viewed as a measure of the willingness of an individual over the proposed treatment period to take part in MDA treatment.

The likelihood of achieving interruption of transmission depends on many factors. One of the key parameters is STH prevalence at baseline (before LF treatment takes place), which is defined by the intrinsic transmission intensity (R_0_). A range of R_0_ values were investigated (R_0_ = 1.6–3.0), representing a range of observed baseline prevalences. Other parameters, such as the density dependence in fecundity, the age-intensity profile and the aggregation parameter, were all fixed as defined in Table 1.

Five hundred simulations were run for each scenario (control arm, intervention arm with fixed coverage and intervention arm with varied coverage) for both *A*. *lumbricoides* and hookworm. Clusters were constructed from two or more “villages” (village size is fixed to 1000 individuals per village) consisting of 2,000 to 4,000 individuals per cluster, which corresponds to the DeWorm3 trial design. In this study, we make the simplifying assumption that there is no migration between villages or clusters and that villages are independent units (a future analysis will examine the consequences of movement and migration). The construction of clusters was repeated 10,000 times. To investigate the effects of increasing coverage levels on achieving elimination in individual clusters, 500 simulations were run where the pre-SAC, SAC and adult MDA coverage levels were varied from 50 to 90% in increments of 10%.

All simulations were run for 50 years, the first 10 years represent the endemic phase where no treatment is given such that a stable equilibrium can be reached. This is followed by the LF treatment phase (4 years) and the STH treatment period (3 years). During the remaining period no treatment is provided, and simulations either achieved interruption of transmission or bounced back towards endemic levels prior to MDA. At year 50, we investigated whether interruption of transmission had been achieved. Throughout this study, the prevalence is measured community-wide unless otherwise stated.

Positive and negative predictive values (PPV/NPV) were calculated (six months after the last round of MDA) based on a range of prevalence thresholds [6]. The PPV and NPV are the proportion of positive and negative outcomes in the trials that are true positive (elimination of transmission is achieved) and true negative results (bounce-back occurs), respectively. When approaching interruption of transmission in a population, the intensity of infections becomes so low that standard diagnostic tools, such as Kato Katz, become less sensitive in detecting parasitic eggs. Therefore, molecular techniques detecting parasite DNA in stool samples are necessary to determine if interruption of transmission has been achieved. We assumed 94% sensitivity for qPCR assays for both *A*. *lumbricoides* and hookworm species [23,25]. Most diagnostic tools for STH species are based on the presence or absence of eggs in a host stool sample. Both species only produce fertilized eggs when a male and female worm are present in the same host. However, unfertilized eggs can be produced by female *A*. *lumbricoides* worms even in the absence of a male. As such, qPCR techniques cannot distinguish between fertilized and unfertilized eggs. Therefore, in this study, a person is found positive for *A*. *lumbricoides* if they have at least one female worm, whilst for hookworm a person is found positive if they have at least one female and one male worm. The PPV and NPV values were calculated six months after the last round of MDA. As the treatment frequency differs between the intervention and control arm, the PPV and NPV values are effectively measured twelve months post MDA cessation in the control arm and six months post MDA cessation for the intervention arm.

## Results

**Fig 1:**
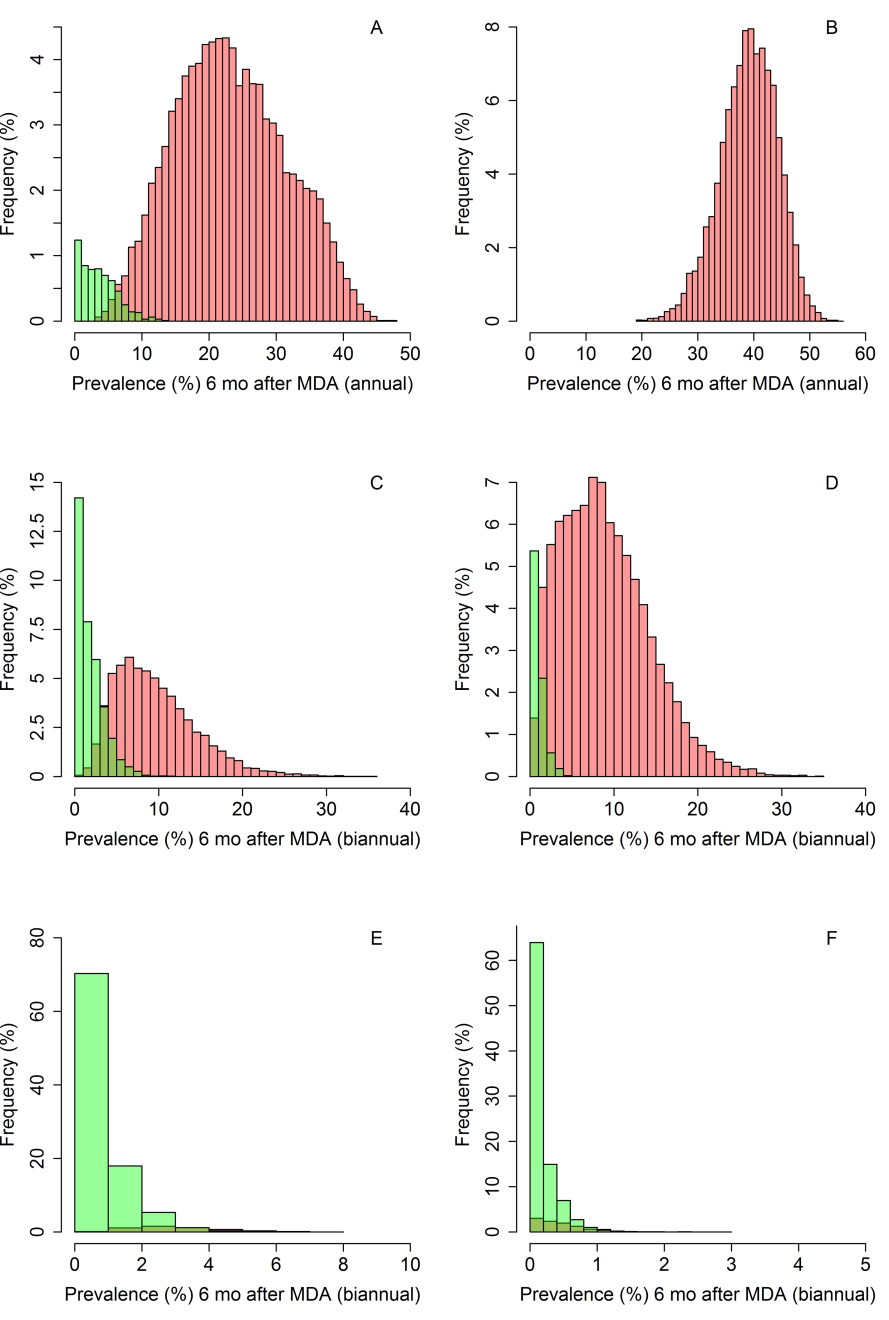
Community-wide prevalence of *A*. *lumbricoides*(A, C and E) and hookworm (B, D and F) for community-based treatment (A and B, coverage is 80% in pre-SAC and SAC and no treatment is provided to adults), intervention arm with a varied coverage (C and D, coverage is 50–90% for pre-SAC and SAC, and 40–80% in adults) and intervention arm with high fixed coverage (E and F, coverage is 90% in pre-SAC and SAC, 80% in adults). Green bars represent the simulations that achieve elimination and the red bars represent those that will eventually bounce back to pre-MDA prevalence levels if no more treatment is applied.

Fig 1A and 1B show the distribution of community-wide prevalence values in the control arm for *A*. *lumbricoides* and hookworm, respectively, six months after the last round of MDA. Interruption of transmission is unlikely in the control arm for both species of STH. For *A*. *lumbricoides*, a small proportion of simulations (6.1%) reached elimination, whilst in the hookworm simulations, none of the control arm simulations achieved interruption of transmission (Fig 1A and 1B and Table 2). Although MDA targeted at pre-SAC and SAC may also have an indirect impact on the prevalence and worm intensity in adults due to a reduced pool of infective stages arising from the treatment of children, it is very unlikely that treating only children will result in interruption of transmission.

**Table 2:** Overview of positive predictive value (PPV) and negative predictive value (NPV) results (values of 1.00 or close to it are desirable) and the proportion of *A*. *lumbricoides* simulations (%) achieving the defined prevalence threshold (community-wide) six months post MDA which do not result in the interruption of transmission.

Prevalence threshold (%)	Control arm PPV/NPV	Control arm Likelihood (%) of prevalence > threshold	Intervention arm (varied) PPV/NPV	Intervention arm (varied) Likelihood (%) of prevalence > threshold	Intervention arm (fixed) PPV/NPV	Intervention arm (fixed) Likelihood (%) of prevalence > threshold
0.5	1.00/0.95	93.89	1.00/0.70	64.72	1.00/0.10	5.01
1	1.00/0.95	93.89	1.00/0.75	64.66	1.00/0.17	4.91
2	1.00/0.96	93.89	0.98/0.83	64.22	0.99/0.36	3.80
3	1.00/0.97	93.89	0.94/0.90	62.57	0.97/0.60	2.26
4	0.99/0.97	93.83	0.87/0.94	58.96	0.96/0.76	1.08
5	0.97/0.98	93.68	0.77/0.97	53.71	0.96/0.85	0.41
6	0.92/0.98	93.35	0.89/0.98	48.04	0.95/0.91	0.10
7	0.84/0.99	93.90	0.62/0.99	43.18	0.95/NaN	0.01
8	0.78/0.996	92.10	0.56/1.00	36.44	0.95/NaN	*

* There were no simulations that had a measured prevalence of 8% or higher.

For the intervention arm, assuming high treatment coverage (90% in pre-SAC and SAC, 80% in adults), the prevalence range is very narrow when measured six months after the last round of MDA (Fig 1E and 1F). Therefore, ruling out the interruption of transmission is challenging due to the low prevalences achieved in all simulated clusters during the six months following the last round of MDA, independent of the eventual outcome (bounce-back or transmission interruption). In the *A*. *lumbricoides* simulations, 99.4% of the clusters have a measured prevalence of less than 5% six months after MDA cessation and only a small proportion of simulations (5%) eventually bounced back to pre-MDA levels. In the hookworm simulations, the range of prevalences six months post-MDA are even narrower. All simulations have a measured prevalence less than 3%, and 90% of the simulations achieve interruption of transmission (Fig 1E and 1F and Tables 2 and 3).

**Table 3:** Overview of positive predictive value (PPV) and negative predictive value (NPV) results and the proportion of hookworm simulations (%) achieving this threshold which do not achieve interruption of transmission.

Prevalence threshold (%)	Intervention arm (varied) PPV/NPV	Intervention arm (varied) Likelihood (%) of prevalence > threshold	Intervention arm (fixed) PPV/NPV	Intervention arm (fixed) Likelihood (%) of prevalence > threshold
0.5	0.93/0.94	91.33	0.94/0.29	4.63
1	0.84/0.97	90.13	0.91/0.58	0.86
2	0.61/0.99	85.63	0.90/NaN	0
3	0.44/1.00	80.11	0.90/NaN	0

If the desired coverage levels are not achieved, the prevalence measured six months post- cessation of MDA shows a much larger range (Fig 1C and 1D). For both *A*. *lumbricoides* and hookworm, the endline prevalence range of the simulated clusters was 0–36%. In this case, it would be possible to differentiate between the likelihood of bounce-back and true interruption of transmission in the immediate time period following MDA cessation. To completely rule out the interruption of transmission would require a negative predictive value (NPV) of 1.00. However, in practice this may be unrealistic and a more reasonable certainty of an NPV value of 0.90 or 0.95 may be applied (90% or 95% certainty of a cluster bouncing-back to pre-treatment levels). An NPV value of 1.00 was achieved at a prevalence threshold of 8% for *A*. *lumbricoides* (Table 2). In the *A*. *lumbricoides* simulations, 36.4% of simulations had a prevalence greater than 8% six months post-MDA when the intervention arm with varied coverage was simulated. Using a 5% prevalence threshold, the NPV value for *A*. *lumbricoides* was 0.95, implying that only a very small proportion of simulations achieved interruption of transmission. For simulations with fixed coverage the NPV was 0.85 for a prevalence threshold of 5%. In this scenario, a threshold of 6% gives more certainty as the NPV value is 0.91.

If the desired coverage levels are met (intervention arm with fixed coverage), for *A*. *lumbricoides* none of the simulated clusters have a prevalence greater than 8% six months post MDA. However, a small proportion of simulations (<5%) still bounce back to endemic levels. A similar result is observed for the *A*. *lumbricoides* control arm, where the prevalence range is very wide and only a small proportion of clusters reach elimination. Only 0.4% of these simulations had a measured prevalence above 8% and achieved interruption of transmission (Table 2). At 8% prevalence, the NPV is greater than 0.99, suggesting that we can predict in which clusters bounce-back is likely to occur with very high confidence. Therefore, a prevalence threshold of 6–8% for *A*. *lumbricoides* would be a good endpoint for detecting failing clusters (those in which bounce-back is likely to occur), but only if there is good evidence that the required coverage levels are not achieved during MDA treatment rounds.

In the hookworm simulations, the NPV was 1.00 when the prevalence threshold was set at 3%. 80% of simulations with varied coverage (intervention arm) achieved this prevalence. However, all simulations of the intervention arm with high, fixed coverage had a measured prevalence less than 3% six months post-cessation of MDA. Therefore, ruling out transmission is not possible for hookworm if the desired coverage levels are achieved. If this is not the case, however, a prevalence of 3% or higher measured six months after the last round of MDA is likely to rule out the interruption of transmission. The hookworm results do not include results from the control arm, as none of these simulations achieved interruption of transmission in these simulations.

To investigate the impact of MDA coverage on the likelihood of failing to reach interruption of transmission, simulations were run for simulation for clusters with coverage levels varying from 50% to 90% in increments of 10% for pre-SAC, SAC and adults (we assumed that the coverage levels for pre-SAC and SAC were the same).

To achieve interruption of transmission in areas where both species (*A*. *lumbricoides*and hookworm) are endemic, a high MDA coverage should be achieved across all age groups. However, if only one species is prevalent in the area, the importance of achieving high MDA coverage in the pre-SAC & SAC population or the adult population depends on the species of STH present. For *A*. *lumbricoides*, achieving high coverage in the pre-SAC and SAC population is vital to achieve interruption of transmission. For example, if the MDA coverage in pre-SAC & SAC is 90% and for adults is 60%, the likelihood of successful transmission interruption is 71.7%, whilst a high coverage in adults (90%) and low coverage in pre-SAC & SAC (60%) gives a lower likelihood of achieving interruption of transmission of 31.3% (Table 4). For hookworm, however, the opposite is true given the observed age intensity profiles for hookworm, with the highest rates of infection in adults. Achieving a high MDA coverage in pre-SAC & SAC (90%) and low adult coverage (60%) results in interruption of transmission in 4.1% of the simulated clusters, whereas if a high adult coverage (90%) and low pre-SAC & SAC coverage (60%) is achieved, all of the simulations achieved interruption of transmission (Table 5).

**Table 4:** The likelihood of achieving interruption of transmission (%) of *A*. *lumbricoides* in a cluster.

	Pre-SAC and SAC coverage (%)					
Adult coverage (%)		50%	60%	70%	80%	90%
	50%	12.3	15.2	22.7	36.9	50.9
	60%	11.5	16.5	28.3	46.8	71.7
	70%	14.7	21.0	33.2	55.5	81.5
	80%	15.0	25.9	43.7	71.8	92.1
	90%	16.8	31.3	52.6	80.5	97.2

**Table 5:** The likelihood of achieving interruption of transmission (%) of hookworm in a cluster.

	Pre-SAC and SAC coverage (%)					
Adult coverage (%)		50%	60%	70%	80%	90%
	50%	<1.0	<1.0	<1.0	<1.0	<1.0
	60%	2.5	4.1	3.7	3.9	4.1
	70%	13.6	15.1	20.4	22.9	22.3
	80%	53.8	69.2	82.6	91.6	91.8
	90%	91.9	100	99.6	100	100

## Discussion

This study highlights the difficulties of discriminating between bounce-back and the interruption of transmission quickly (within six months) following six rounds of MDA in a community trial, even with frequent, high coverage treatment of all age classes. These findings are in line with those described in Truscott et al., (2017) [21]. The prevalences in successful clusters (where transmission is eliminated) and unsuccessful clusters (where transmission persists) are close in value to each other in the six months immediately following cessation of MDA (or twelve months in the case of the control arm). However, MDA coverage levels over three consecutive years of treatment are a good indicator of the eventual outcome. If trials such as DeWorm3 achieve the targeted 80–90% coverage of all age groups, the prevalence range will be tight in the intervention clusters six months post MDA. The coverage required to achieve elimination depends on the baseline prevalence (transmission intensity as measured by R_0_) in a community. Determining whether or not transmission interruption has occurred is difficult unless sufficient time has passed following cessation of MDA to determine whether bounce-back is likely to occur[21]. An improved understanding of the intrinsic transmission potential in different settings and well-monitored programmes/studies are needed to understand why the interruption of transmission may be achieved in some environments and not in others.

The DeWorm3 trials take place in areas with sustained LF control programmes, including MDA coverage community-wide. In areas without LF programmes it may be possible to build upon existing STH programmes followed by community-wide intensified MDA programmes. However, the successes will depend on the dominant species and the age-intensity profiles. As children typically suffer more from *A*. *lumbricoides* than adults and the opposite is generally found for hookworm, a strong LF programme is more important in areas where hookworm is the dominant species. However, to increase the likelihood of achieving interruption of transmission in areas without a sustained LF programme, the STH programme could be intensified, by increasing the number of rounds or treatment duration. The duration of such a programme or required rounds of treatment depends on the transmission intensity, age intensity profiles and clustering, which should always be considered on a case by case basis.

It is essential to define the targeted population by well-designed census methods in a trial, or other community-based treatment programmes, to accurately measure the achieved coverage and individual compliance to multiple rounds of treatment. If the population size is underestimated then the true coverage is lower than the measured coverage, effectively leaving a proportion of the population untreated. Achieving high MDA coverage (> 75%) has been shown to be difficult in some areas [30]. This may be because parts of the population live in difficult to reach areas, children from some households may not attend school, conflicts may be present in an area, or due to insufficient adherence to treatment. Even though albendazole has very few side effects, some individuals may be apprehensive due to side effects arising in other MDA programmes using, for example, praziquantel to treat schistosome infections. Moreover, the WHO recommends not treating women during the first trimester of their pregnancy [2]. As there is often uncertainty of the timing of the start of pregnancy, women of child-bearing age often do not receive, or take, anthelmintic drugs. If there are indications that the achieved treatment coverage is lower than 80% in any one age grouping (children or adults), the prevalence threshold for *A*. *lumbricoides* should be between 6–8% and around 3% for hookworm to suggest possibly stopping the trial and continuing with standard of care MDA to control morbidity.

The sensitivity of qPCR is poorly defined at present in low prevalence areas since it is not widely used. For this analysis we have assumed 94% sensitivity [25]. If the sensitivity is greater, the endpoint prevalence threshold for stopping will also be higher as the precision around the final prevalence estimate will be greater. However, if the sensitivity of qPCR diagnostic is lower, it will be more difficult to differentiate between clusters that are likely to achieve interruption of transmission and clusters that are likely to fail. Quantitative scores from qPCR tests depend on the worm burden of an individual [25,31]. The higher the worm burden the more sensitive any diagnostic tool will be.

In this study, the village size was fixed to 1,000 individuals and clusters were constructed of 2,000–4,000 individuals (in line with the DeWorm3 trial). Previous research that investigated the impact of the number of villages within a cluster showed that an increase in the number of villages in a cluster reduces the likelihood of achieving interruption of transmission at a cluster level [4]. However, the villages in the current study were selected at random from the simulated villages, meaning that transmission intensities (or baseline prevalences) showed considerable variability. In the field, spatial patterns are observed where some geographical areas have a low baseline prevalence whilst others have high baseline prevalences. The causes for the observed spatial heterogeneity are not well understood but environmental and socio-demographic factors will be important. If a cluster is constructed from multiple high prevalence villages (measured at baseline), achieving high coverage levels becomes increasingly important.

In this study we assumed that the village sizes were fixed to 1000, however, in the DeWorm3 trials the village sizes differ largely. In some cases, a village can contain over 10,000 individuals and these villages are divided into several clusters. Transmission dynamics have been mostly studied within smaller villages and found that the village is the most appropriate epidemiological unit [32]. However, in large villages it may become unrealistic to assume a single independent transmission unit. The DeWorm3 trials will collect data to investigate the transmission dynamics in larger villages.

Migration patterns were not included in this study; however, migration reduces the likelihood of achieving interruption of transmission if infected individuals do not receive treatment before returning to their home village [6]. However, these migration patterns have not been studied in detail for STH infections. The DeWorm3 study will collect data relating to this issue and will consequently be included in the mathematical models.

The immediate determination of whether transmission interruption has failed is therefore difficult, given the narrow range of prevalences observed post-treatment and expected measurement errors in determining the prevalence. It is important that sufficient time has passed following cessation of MDA when measurements of prevalence are used to determine whether bounce back is likely to occur. Simulations suggest that it would not be desirable to make a judgement regarding the probability of bounce-back early (such as six months post-MDA) unless coverage levels lower than 80% are consistently recorded. Assessment of prevalence two years post-cessation of MDA provides a high degree of certainty in determining the likelihood of transmission interruption.
